# Sensory processing sensitivity levels in patients with disorders of gut–brain interaction: a propensity score-matched cross-sectional study

**DOI:** 10.3389/fmed.2026.1674680

**Published:** 2026-03-24

**Authors:** Dingjiang Peng, Yijun Zhang

**Affiliations:** 1 Guangzhou University of Chinese Medicine, Guangzhou, China; 2 General Hospital of Southern Theater Command, Guangzhou, China

**Keywords:** discriminative capacity, disorders of gut–brain interaction, highly sensitive persons, propensity score matching, sensory processing sensitivity

## Abstract

**Background:**

Although sensory processing sensitivity (SPS) has been extensively studied in the context of psychological health, its potential association with disorders of gut–brain interaction (DGBI) remains poorly understood.

**Objective:**

This study used the Chinese version of the Highly Sensitive Person (HSP) Scale to assess sensory processing sensitivity (SPS) levels and the prevalence of highly sensitive persons (HSPs) in a DGBI cohort. Using propensity score matching (PSM), it aimed to compare these metrics between DGBI patients and healthy controls and to evaluate the discriminative capacity of SPS for DGBI. Additionally, SPS and HSP distribution were compared across DGBI subgroups categorized by upper versus lower gastrointestinal (GI) symptom predominance.

**Methods:**

SPS levels were assessed using the validated Chinese Highly Sensitive Person Scale (HSPS-C). Covariate balance was achieved through 1:1 PSM. Intergroup disparities were examined using independent samples *t*-tests and *χ^2^* analyses, supplemented by the binary logistic regression analysis and the receiver operating characteristic (ROC) curve analysis.

**Results:**

Post-PSM analysis (254 matched pairs) revealed significantly higher total SPS scores in the DGBI group versus controls (*p* < 0.001), with a substantially greater HSP prevalence (42.5% vs. 17.3%; *p* < 0.001). Subgroup analysis showed no statistically significant differences in SPS levels or HSP distribution between patients with upper and lower gastrointestinal DGBI (*p* > 0.05). Ease of excitation (EOE; OR = 1.403, 95% CI: 1.110–1.773) and depth of processing (DOP; OR = 1.315, 95% CI: 1.044–1.657) emerged as independent DGBI risk factors. ROC analysis showed a moderate discriminative capacity for DGBI identification (AUC = 0.705, 95% CI: 0.660–0.750).

**Conclusion:**

Elevated SPS levels are significantly associated with DGBI. However, the subgroup analysis revealed no significant differences in SPS levels or HSP distribution between patients with upper and lower gastrointestinal DGBI. Early identification of HSP characteristics may nevertheless facilitate personalized interventions, supporting the integration of SPS assessment into DGBI diagnostic and management frameworks.

## 1 Introduction

Disorders of gut–brain interaction (DGBI) are a group of disorders classified by gastrointestinal symptoms related to any combination of the following: a motility disturbance, visceral hypersensitivity, altered mucosal and immune function, altered gut microbiota, and altered central nervous system (CNS) processing (1). As per Rome IV criteria, DGBI diagnosis requires symptom persistence ≥6 months with active manifestations in the preceding 3 months (2). Epidemiological surveillance has indicated that the global prevalence of DGBI is approximately 40% (30% in China), demonstrating significant female predominance and substantial quality-of-life impairment compared to healthy populations (3). Based on the primary symptom regions and characteristics defined in the Rome IV criteria, DGBI are categorized into several major groups. The most prevalent subtypes include irritable bowel syndrome (IBS), characterized by recurrent abdominal pain related to defecation or associated with changes in bowel habits (subclassified as IBS-C, IBS-D, IBS-M, or IBS-U), and functional dyspepsia (FD), characterized by postprandial fullness, early satiation, or epigastric pain/burning. Other common DGBI include functional constipation, functional diarrhea, and functional abdominal bloating/distention (4).

The pathophysiology of DGBI remains incompletely understood. Current Rome IV nosology classifies DGBI as disorders of gut–brain interaction (DGBI), with core mechanisms involving dysregulated central nervous system processing of visceral afferent signals (2). Clinical investigations have revealed visceral hypersensitivity in 33–90% of IBS patients (5), suggesting abnormal sensory processing as a key pathophysiological mechanism. However, individual differences in central sensory modulation patterns remain largely unexplored.

Sensory processing sensitivity (SPS), a genetically influenced temperament trait first conceptualized by Aron et al. (6) in 1997, reflects differential responsiveness to environmental stimuli. Highly sensitive persons (HSPs) constitute 20–30% of general populations (7). The depth-of-processing characteristic of HSP manifests as heightened reactivity to internal/external stimuli (e.g., pain and noise) and amplified psychophysiological responses to adverse stimuli (8, 9). Research has shown that SPS exhibits a dual-edged nature in individual development. Compared with the general population, HSPs exhibit heightened susceptibility to psychological symptoms such as anxiety and depression when exposed to adverse developmental environments (10). Conversely, they tend to outperform their less sensitive counterparts in supportive contexts (11). Notably, HSPs exhibiting psychological symptoms derive greater therapeutic benefits from behavioral interventions (12). As research advances, emerging evidence suggests that SPS not only correlates with psychological health concerns but may also influence somatic health through amplified somatic symptom awareness. Iimura et al.’s (13) epidemiological survey of 863 Japanese adults showed positive correlations between SPS levels and gastrointestinal symptoms (diarrhea and abdominal pain), suggesting SPS-mediated brain–gut axis dysregulation in DGBI pathogenesis. Nevertheless, systematic investigations into SPS-DGBI causality and underlying neurobiological mechanisms remain absent.

### 1.1 Research rationale and hypotheses

Despite progress in DGBI research, the role of psychological factors—particularly sensory processing patterns—in disease progression remains unclear. Existing studies focus on peripheral neural sensitization or gut microbiota dysbiosis, neglecting the hypothesis that central nervous system hyperreactivity exacerbates visceral hypersensitivity via gut–brain axis dysregulation. Based on this, and given that SPS is fundamentally a trait of central nervous system processing, we hypothesized that: (1) DGBI patients exhibit significantly higher SPS levels and HSP prevalence than healthy controls and (2) this association would be consistent irrespective of the primary symptomatic region, with no significant differences in SPS levels or HSP distribution between patients with upper and lower gastrointestinal DGBI. Using PSM to control confounders, this study aimed to validate these hypotheses and assess the diagnostic utility of SPS for DGBI, providing a foundation for early clinical intervention strategies.

## 2 Materials and methods

### 2.1 Diagnostic and assessment tools

#### 2.1.1 Highly sensitive person scale

This study used the validated Chinese version of the Highly Sensitive Person Scale (HSPS-C) adapted by Zhang et al. (14). This instrument originated from Aron et al.’s (6) original HSPS, undergoing rigorous cross-cultural adaptation through Brislin’s translation model, which involved forward translation, synthesis, back-translation, expert panel review, and pretesting. The adapted HSPS-C has shown strong psychometric properties. The HSPS-C comprises 27 items (e.g., “I am generally more sensitive to pain”) organized into six clinically relevant dimensions: 1. *emotional reactivity*: responsiveness to affective stimuli; 2. *low sensory threshold*: hypersensitivity to sensory input; 3. *ease of excitation (EOE)*: propensity to become overaroused; 4. *esthetic sensitivity*: responsiveness to subtle environmental cues; 5. *punishment sensitivity*: anticipatory anxiety toward negative outcomes; 6. *depth of processing (DOP)*: tendency for elaborate cognitive processing.

The responses were recorded using a 7-point Likert-type scale ranging from 1 (“*not at all*”) to 7 (“*extremely*”). The total score was computed by summing all item responses, theoretically ranging from 27 (minimum sensitivity) to 189 (maximum sensitivity), with higher scores indicating greater sensory processing sensitivity.

#### 2.1.2 Hospital anxiety and depression scale

The Hospital Anxiety and Depression Scale (HADS) was used to assess symptoms of anxiety and depression in the study population. The HADS comprises two subscales: one for anxiety (HADS-A) and one for depression (HADS-D), each containing seven items. Items are rated on a 4-point Likert-type scale ranging from 0 (not at all) to 3 (very much), with higher scores indicating greater severity of symptoms. For each subscale, a score of ≥11 indicates clinically significant symptoms (“definite case”), scores of 8–10 indicate “probable cases,” and scores of 0–7 indicate “non-cases.” The HADS is a well-validated and widely used instrument for screening anxiety and depression (15).

#### 2.1.3 Examination equipment

All participants in the DGBI group underwent a standardized diagnostic workup to exclude organic diseases and refine phenotypic classification. The procedures and tools used were as follows: Gastroscopy and Colonoscopy – Equipment: High-definition electronic video endoscopes (Gastroscope: Olympus GIF-HQ290; Colonoscope: Olympus CF-HQ290I). Procedure standardization: Narrow-Band Imaging (NBI), a form of digital chromoendoscopy, was routinely used to enhance mucosal detail. Supplementary Digestive Physiology Tests (performed in a subset of participants) – Indication: Administered based on clinician judgment for patients with symptoms highly suggestive of specific motility or sensory disorders (e.g., refractory heartburn, dysphagia, and chronic constipation with straining). Equipment and Procedures – High-Resolution Esophageal Manometry (HRM): Solid-state manometric catheter (ManoScan™ system, Medtronic) was used to assess esophageal motility patterns. 24-h pH-Impedance Monitoring: Combined pH-impedance catheter (Digitrapper™, Medtronic) was used to quantify gastroesophageal reflux (acid and non-acid). Purpose: Results supported or excluded diagnoses such as major esophageal motility disorders and reflux hypersensitivity, thereby increasing diagnostic precision for functional syndromes.

### 2.2 Study population

This cross-sectional study recruited two distinct cohorts: a case group of consecutive DGBI patients (meeting Rome IV diagnostic criteria) visiting the Gastroenterology Clinic at the General Hospital of Southern Theater Command (Guangzhou) between January 2024 and August 2024, and a control group of asymptomatic individuals without gastrointestinal disorders, systematically enrolled from the hospital Health Management Center during the identical recruitment period.

Inclusion criteria for the case group were as follows: 1. Aged 18–60 years; 2. Met the Rome IV diagnostic criteria for DGBI; 3. No history of gastrointestinal surgery; 4. Provided written informed consent; 5. Completed the questionnaires within 8–20 minutes; 6. No current or previous use of psychotropic medications, and confirmed absence of anxiety or depressive states using the Hospital Anxiety and Depression Scale (HADS) prior to enrollment; 7. Negative result for the Helicobacter pylori test; 8. Organic lesions were excluded through a standardized diagnostic workup, which included: Endoscopic Procedures: All participants underwent both electronic gastroscopy and colonoscopy, performed by a team of three designated experienced endoscopists to ensure consistency. Narrow-band imaging (NBI) was routinely used to enhance mucosal visualization. Upper Endoscopy Assessment: For gastroscopy, the Kyoto classification of gastritis was used to score mucosal findings, with specific attention paid to NBI patterns such as the regular arrangement of collecting venules (RAC) to exclude atrophic gastritis. Colonoscopy Quality Control: Colonoscopy was required to reach the cecum, and the Boston Bowel Preparation Scale (BBPS) score was required to be ≥2 in all segments. Laboratory Investigations: Routine blood tests, stool tests (including occult blood and calprotectin), and abdominal ultrasonography were performed to exclude metabolic, inflammatory, or structural abnormalities.

Exclusion criteria for the case group were as follows: 1. Organic diseases (e.g., inflammatory bowel disease) confirmed by endoscopic or pathological examination; 2. History of gastrointestinal surgery; 3. Age < 18 or > 60 years; 4. Refusal to participate or withdrawal from the study; 5. Questionnaire completion time < 8 minutes or > 20 minutes; 6. Psychiatric disorders (e.g., depression and anxiety) or current/previous use of psychotropic medications; 7. Positive result for the Helicobacter pylori test.

Inclusion criteria for the control group were as follows: 1. No primary gastrointestinal symptoms (assessed by a Gastrointestinal Symptom Rating Scale [GSRS] total score < 5); 2. Aged 18–60 years; 3. Provided written informed consent; 4. Completed the questionnaires within 8–20 minutes; 5. No current or previous use of psychotropic medications and confirmed to be free of psychiatric disorders by evaluation in the Department of Psychology. Exclusion criteria for the control group were the same as those for the case group.

### 2.3 Ethics approval and consent to participate

This study received approval from the Ethics Committee of General Hospital of Southern Theater Command (Ethics No: NZLLKZ2025004) and adhered to the principles outlined in the Declaration of Helsinki. Prior to distributing the questionnaires, informed consent was obtained from all participants included in the study.

Consent for publication.

### 2.4 Propensity score matching

To address potential confounding from baseline characteristics (age, sex, and education), we implemented propensity score matching (PSM) using the MatchIt package (version 4.5.0) in R 4.4.2. The propensity score was estimated using logistic regression with DGBI diagnosis as the dependent variable (case = 1, control = 0), incorporating three covariates: age (continuous, years), sex (male = 0, female = 1), and educational attainment level (≤high school = 0, >high school = 1). A 1:1 nearest-neighbor matching algorithm without replacement was executed with a caliper width of 0.02 standard deviations of the propensity score logit. Post-matching balance was evaluated using standardized mean differences (SMD), with SMD values <0.1 indicating adequate covariate balance (16).

### 2.5 Sample size calculation

The study was designed as a cross-sectional investigation to compare whether there are differences in SPS levels between DGBI patients and healthy populations. Based on previous experience and a review of relevant literature, the estimated standard deviation was 1.17, with requirements for a two-tailed test, *α* = 0.05, and a margin of error of 0.50. The sample size was calculated using the following formula, resulting in a sample size of *N* = 230. Therefore, this study required a minimum of 230 participants as the study subjects.

Formula: $n={\left(\frac{z_{\alpha^{*\sigma }}}{\delta }\right)}^{2}$

### 2.6 Handling of incomplete data

To ensure data integrity, the following procedures were implemented during questionnaire collection:

#### 2.6.1 Completeness control

Pre-collection Protocol: Researchers provided standardized on-site instructions emphasizing the necessity of completing all items.

Real-time Verification: Questionnaires were immediately reviewed post-collection. Participants with missing entries were re-contacted to complete them.

Time Threshold Validation: Based on pre-trial results (mean completion time: 15 min), questionnaires completed in <8 min or >20 min were excluded to minimize invalid responses from rushed or interrupted participation.

#### 2.6.2 Management and imputation of missing values

Among the 897 valid questionnaires retained for analysis, the missing value rate was negligible (<0.1%), primarily affecting basic demographic variables (e.g., age and sex). The following strategies addressed residual missing data: Listwise Deletion: Questionnaires with >20% missing items per case were excluded from subsequent analyses. Mean Imputation: Sporadic missing numerical variables (e.g., age) were replaced with the variable’s mean to preserve dataset completeness.

### 2.7 Statistical analysis

Data were managed through dual independent entry with cross-verification. Analyses were conducted using IBM SPSS 26.0 and R 4.4.2. Continuous variables were assessed for normality using the Shapiro–Wilk test. Normally distributed data were presented as mean ± SD and analyzed using independent *t*-tests; non-normally distributed data were reported as M (P25, P75) and analyzed using the Mann–Whitney *U* tests. Categorical variables were summarized as frequencies (%) and compared using the χ^2^ or Fisher’s exact tests. The common method bias test was used to evaluate data validity. Binary logistic regression was performed to identify risk factors, and receiver operating characteristic (ROC) curve analysis assessed the model’s discriminative performance. A two-tailed test was used with a significance level of *α* = 0.05, and *p* ≤ 0.05 was considered statistically significant.

## 3 Result

### 3.1 Baseline characteristics before and after matching

**Figure 1:**
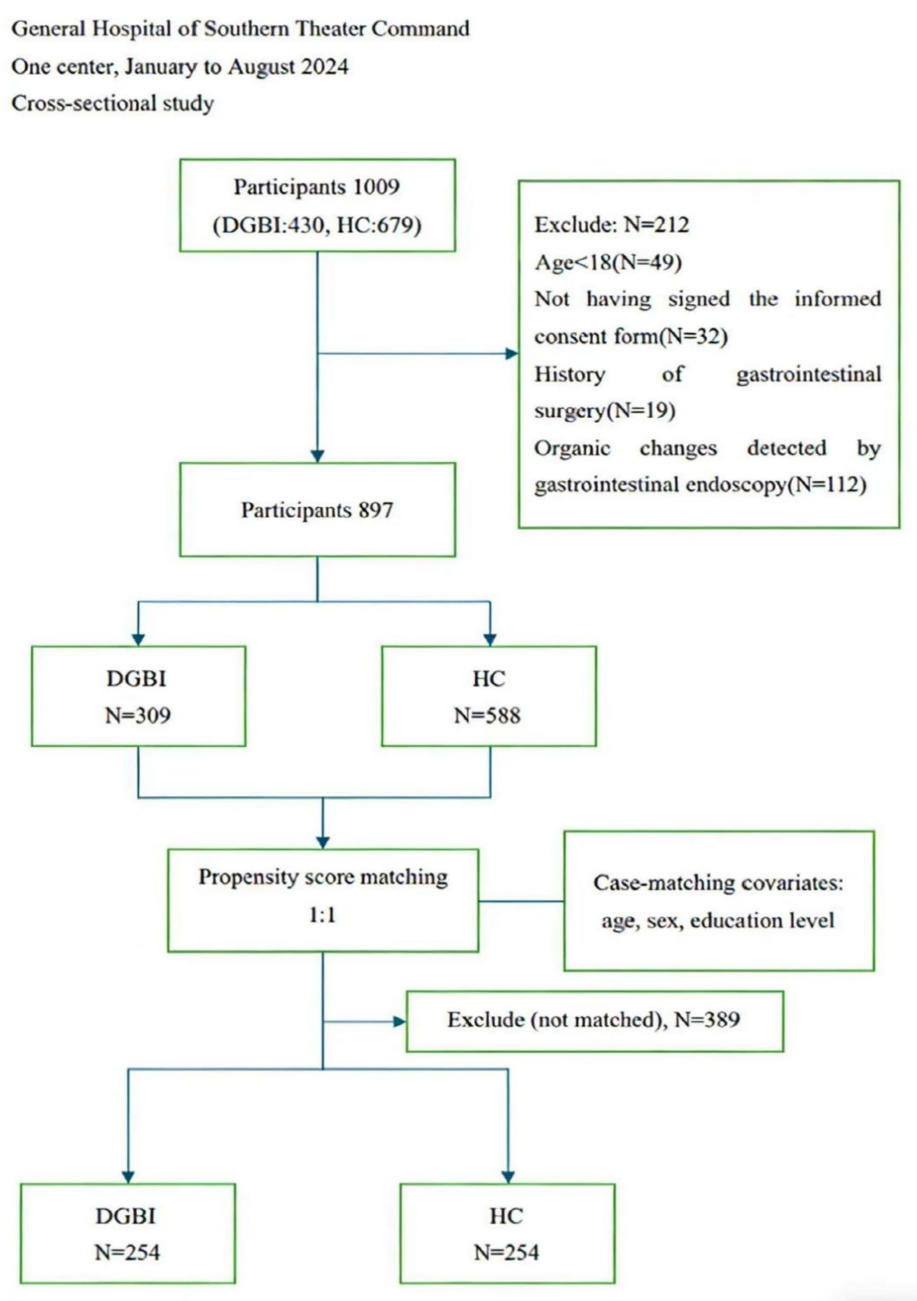
Flowchart of screening and propensity score matching. DGBI: disorders of gut–brain interaction, HCs: healthy controls.

The investigation initially screened 1,009 potential participants. Following rigorous application of eligibility criteria, 897 subjects (male: *n* = 534; female: *n* = 363; median age: 29 years [interquartile range (IQR): 25–35]) were included in the final analytical cohort, comprising 309 DGBI patients and 588 healthy controls (Figure 1). Pre-matching analysis identified significant intergroup differences in sex, age, and education level (all *p* < 0.05). Post-matching analysis revealed no statistically significant differences in sex, age, or educational attainment between the two groups, with standardized mean differences (*SMD*) < 0.1, indicating sufficient covariate balance (Table 1).

**Table 1:** Comparison of baseline characteristics between case and control groups before and after propensity score matching.

	Unmatched					Matched				
	DGBI (*N* = 309)	HC (*N* = 588)	*Z/χ^2^*	*p*	*SMD*	DGBI (*N* = 254)	HC (*N* = 254)	*Z/χ^2^*	*p*	*SMD*
Age	28 (24,34)	29.5 (25,35)	3.11	0.002	0.151	28 (24,33)	28 (24,33)	0.52	0.603	0.071
Sex			11.76	0.001	0.241			0.39	0.534	0.055
Male (0)	160	374				140	144			
Female (1)	149	214				125	121			
Educational attainment			5.89	0.019	0.170			0.01	0.929	0.008
High school or below (0)	142	221				109	110			
Associate degree or above (1)	167	367				145	144			

### 3.2 Distribution of DGBI subtypes and classification into broad symptom groups

**Figure 2:**
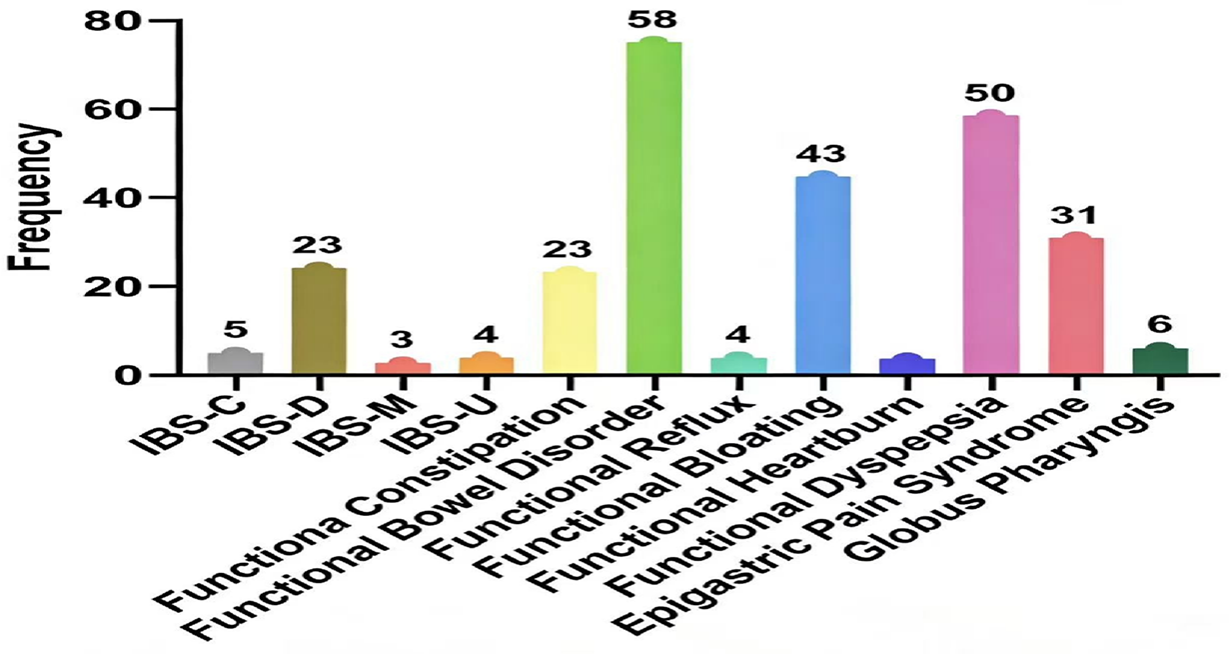
Distribution of DGBI subtypes after matching.

**Figure 3:**
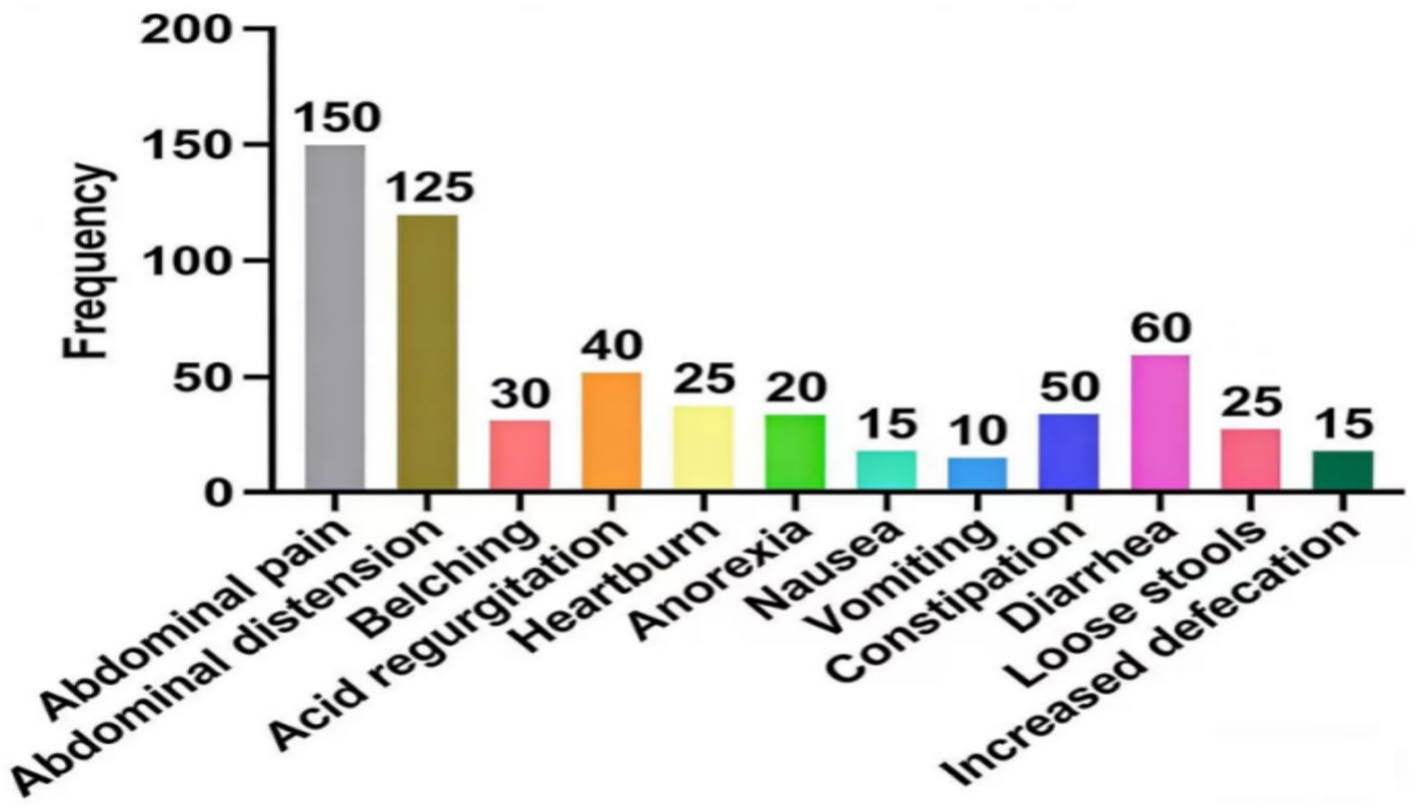
Distribution of clinical symptoms after matching.

According to the Rome IV diagnostic criteria, 254 patients with disorders of gut–brain interaction (DGBI) after propensity score matching were further categorized into specific DGBI subtypes. The diagnosis of each subtype strictly adhered to the Rome IV classification system, based primarily on symptom location, nature, and duration. As shown in Figure 2, functional bowel disorders (FBD) were the most prevalent, with 58 cases (22.8%), followed by functional dyspepsia (FD) with 50 cases (19.7%). Irritable bowel syndrome (IBS), functional bloating, and epigastric pain syndrome (EPS) accounted for 35 (13.8%), 43 (16.9%), and 31 (12.2%) cases, respectively. Another 37 patients (14.6%) met the Rome IV criteria but were not classified into the above major subtypes. The core clinical manifestations in this patient cohort were predominantly abdominal pain and bloating. The detailed distribution of symptom spectra is illustrated in Figure 3.

### 3.3 Common method bias test

The study exclusively used self-reported data from participants, which inherently carries risks of common method bias. To mitigate this potential confound, we implemented procedural safeguards, including anonymous measurement protocols.

The unmeasured latent method factor control (ULMC) approach was used to statistically assess common method variance. This analysis proceeded through two sequential phases: (1) six-factor model construction, developed according to Zhang et al.’s cross-cultural validation framework, and (2) bifactor model extension, which incorporated a common method factor into the established six-factor structure.

**Figure 4:**
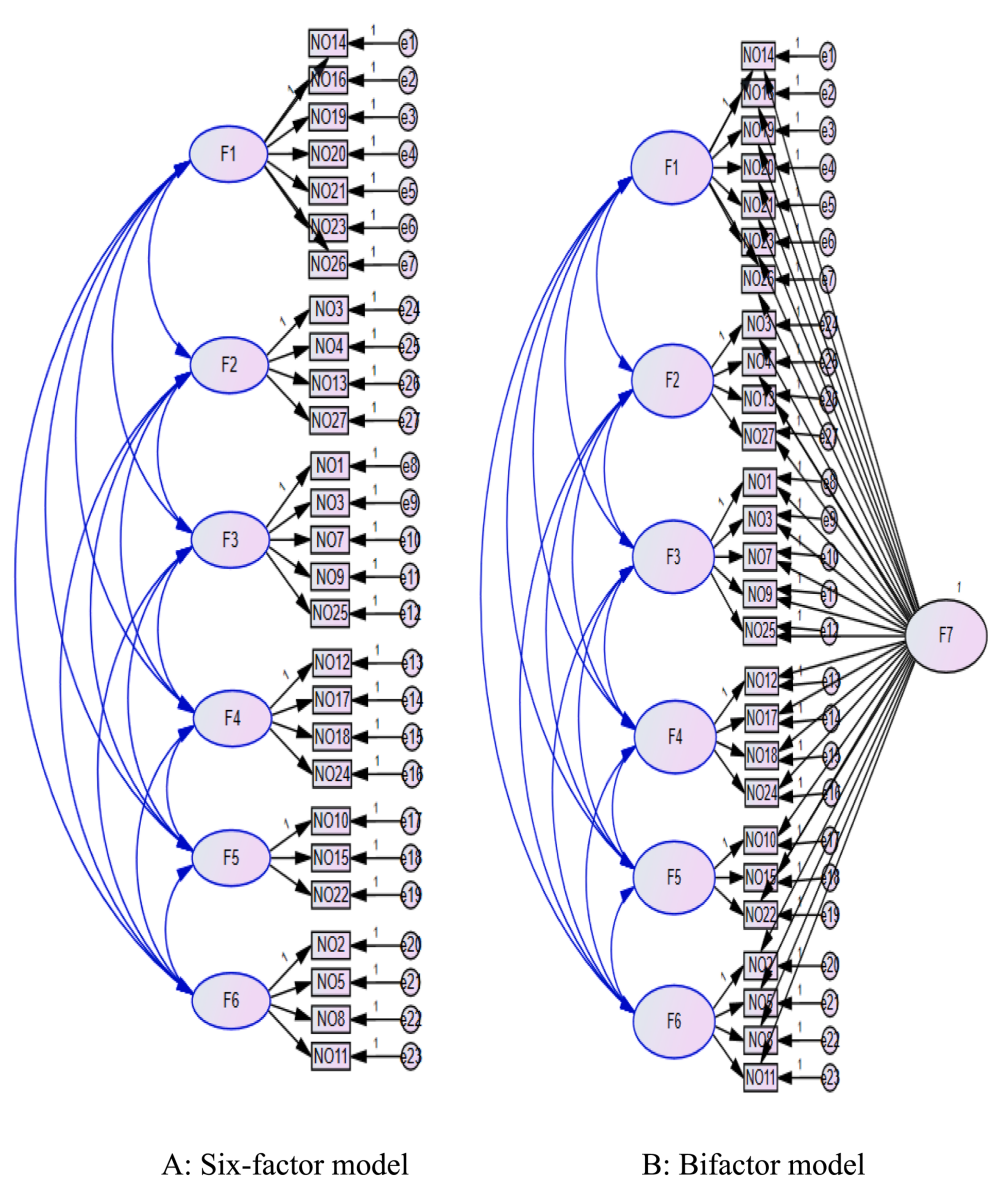
Panel **(A)** presents the six-factor model of the Chinese Highly Sensitive Person Scale (HSPS-C), where F1 to F6 represent the six dimensions: emotional reactivity (ER), low sensory threshold (LST), ease of excitation (EOE), aesthetic sensitivity (AES), punishment sensitivity (PS), and depth of processing (DOP). The numbers (e.g., NO14, NO16) indicate the corresponding items of the HSPS-C. Panel **(B)** shows the bifactor model, which includes a general factor (F7, HSP) in addition to the six specific factors.

The results indicated that the changes in RMSEA between the two models did not exceed 0.05, and the changes in CFI and TLI were less than 0.1 (17). Therefore, the common method bias did not significantly impact the results of this study (Table 2 and Figure 4).

**Table 2:** Comparison of fit indices between the six-factor model and the two-factor model.

Factor model	χ2/df	RMSEA	GFI	AGFI	CFI	TLI	NFI
Six-factor	3.424	0.069	0.862	0.831	0.898	0.884	0.862
Bifactor	3.233	0.066	0.872	0.843	0.906	0.893	0.870
△	0.191	0.003	0.010	0.012	0.008	0.009	0.008

The common method bias analysis revealed acceptable metric stability between comparative models, with △RMSEA≤0.05 and △CFI/△TLI ≤0.1. The absence of significant deterioration in model fit indices indicates no substantial common method bias in the dataset.

### 3.4 Psychometric properties of HSPS-C after post-matching

#### 3.4.1 Reliability analysis

Reliability analysis revealed excellent internal consistency for the HSPS-C, with a Cronbach’s *α* coefficient of 0.950 (>0.9), confirming strong scale reliability (Table 3).

**Table 3:** Reliability indices of the HSPS-C.

	HSPS-C	ER	LST	EOE	AES	PS	DOP
Cronbach’s α coefficient	0.950	0.892	0.826	0.774	0.719	0.695	0.810

The Chinese Highly Sensitive Person Scale (HSPS-C) exhibited strong reliability with a Cronbach’s α of 0.950.

#### 3.4.2 Structural validity analysis

Confirmatory factor analysis (CFA) validated the six-factor structure, with all fit indices meeting the established thresholds (18): CFI = 0.898, TLI = 0.884, RMSEA = 0.069. These psychometric evaluations confirm robust construct validity of the HSPS-C (Table 4).

**Table 4:** Model fit indices of HSPS-C.

Factor model	*χ^2^*/df	RMSEA	TLI	NFI	CFI
Six-factor	3.424	0.069	0.884	0.862	0.898
Reference range	<5.000	<0.080	>0.800	>0.800	>0.800

χ^2^, chi-square; RMSEA, root mean square error of approximation; TLI, Tucker–Lewis index; NFI, normed fit index; CFI, comparative fit index. Structural validity was supported by robust model fit indices: χ^2^/df, RMSEA, NFI, TLI, and CFI all met the recommended thresholds. These findings collectively validate the psychometric robustness of the HSPS-C.

#### 3.4.3 Convergent and discriminant validity

Analyses of convergent and discriminant validity for the HSPS-C were conducted using R version 4.4.2 with RStudio. The standardized factor loadings for each item on its respective factor are presented in Table 1. All item loadings ranged from 0.594 to 0.858, exceeding the recommended threshold of 0.50, indicating that each item effectively represents its intended latent construction. Convergent validity was further assessed using average variance extracted (AVE) and composite reliability (CR). The AVE values for the six factors ranged from 0.514 to 0.654, all above the recommended standard of 0.50. The CR values ranged from 0.812 to 0.918, all exceeding the recommended threshold of 0.70. These results reveal that the scale possesses good convergent validity and internal consistency reliability (Table 5).

**Table 5:** Results of convergent validity analysis.

Item	Factor	Factor loadings	CR	AVE	*P*
No. 14	ER	0.795	0.92	0.61	***
No. 16	ER	0.801			***
No. 19	ER	0.778			***
No. 20	ER	0.738			***
No. 21	ER	0.806			***
No. 23	ER	0.802			***
No. 26	ER	0.737			***
No. 1	LST	0.721	0.88	0.59	***
No. 6	LST	0.630			***
No. 7	LST	0.826			***
No9	LST	0.822			***
No. 25	LST	0.834			***
No. 3	EOE	0.830	0.86	0.6	***
No. 4	EOE	0.787			***
No. 13	EOE	0.801			***
No. 27	EOE	0.668			***
No. 10	AES	0.784	0.84	0.64	***
No. 15	AES	0.810			***
No. 22	AES	0.807			***
No. 12	PS	0.665	0.82	0.53	***
No. 17	PS	0.816			***
No. 18	PS	0.594			***
No. 24	PS	0.809			***
No. 2	DOP	0.668	0.88	0.64	***
No. 5	DOP	0.820			***
No. 8	DOP	0.837			***
No. 11	DOP	0.858			***

***p < 0.001.

Discriminant validity of the scale was assessed by comparing the square root of each factor’s average variance extracted (AVE) with its correlations with other factors, as shown in Table 3. The square roots of the AVE for all factors (ranging from 0.728 to 0.809) exceeded the corresponding inter-factor correlation coefficients (ranging from 0.517 to 0.768), satisfying the Fornell–Larcker criterion and indicating good discriminant validity among the six factors. Furthermore, all inter-factor correlations were below the critical threshold of 0.85, further confirming the distinctiveness between factors. The moderate correlations among factors suggest that they are conceptually related yet sufficiently independent, which is consistent with the multidimensional theoretical framework underlying the highly sensitive person construct (Table 6).

**Table 6:** Results of discriminant validity analysis.

	ER	LST	EOE	AES	PS	DOP
ER	**0.781**					
LST	0.759**	**0.768**				
EOE	0.750**	0.745**	**0.775**			
AES	0.590**	0.576**	0.538**	**0.800**		
PS	0.581**	0.540**	0.517**	0.654**	**0.728**	
DOP	0.714**	0.760**	0.741**	0.620**	0.579**	**0.800**

**p < 0.01. The boldfaced values on the diagonal represent the square roots of AVE, while the off-diagonal values indicate inter-factor correlation coefficients.

### 3.5 Post-matching demographic comparisons

Post-matched demographic characteristics were analyzed using *χ^2^* tests for categorical variables and independent t-tests for continuous variables. Significant intergroup differences were observed in BMI (*t* = 2.11, *p* = 0.035), dietary habits (*χ^2^* = 6.19, *p* = 0.013), and alcohol consumption (*χ^2^* = 24.21, *p* < 0.001) between DGBI patients and HCs. However, no statistically significant difference was observed in smoking status between the groups (*χ^2^* = 1.22, *p* = 0.269) (Table 7).

**Table 7:** Comparison of baseline characteristics between the groups post-matching.

	DGBI (*N* = 254)	HC (*N* = 254)	*t*/*χ^2^*	*p*
BMI	23.26 ± 2.96	22.77 ± 2.14	2.11	0.035
Dietary habits			6.19	0.013
High-fat diet (0)	135	107		
Low-fat diet (1)	119	147		
Alcohol			24.21	<0.001
No (0)	208	243		
Yes (1)	46	11		
Smoking			1.22	0.269
No (0)	181	192		
Yes (1)	73	62		

### 3.6 Analysis of SPS level differences

To evaluate SPS differences between DGBI patients and HCs, independent *t*-tests were performed with total SPS scores as the dependent variable. The analysis revealed significantly higher total HSPS-C scores in the DGBI group than in the HC group (*t* = 8.68, *p* < 0.001). Furthermore, all SPS subdimensions EOE and DOP showed elevated scores in DGBI patients (all *p* < 0.001; Table 8).

**Table 8:** Post-matching comparative analysis of SPS between DGBI patients and HCs.

	All (*N* = 508)	HC (*N* = 254)	DGBI (*N* = 254)	*t*	*p*
HSPS-C	3.91 ± 1.24	3.46 ± 1.25	4.36 ± 1.06	8.68	<0.001
ER	3.89 ± 1.45	3.42 ± 1.45	4.35 ± 1.29	7.66	<0.001
LST	3.62 ± 1.53	3.15 ± 1.49	4.10 ± 1.41	7.38	<0.001
EOE	3.54 ± 1.47	3.00 ± 1.38	4.08 ± 1.34	8.94	<0.001
AES	4.45 ± 1.43	4.14 ± 1.53	4.77 ± 1.25	5.09	<0.001
PS	4.21 ± 1.30	3.93 ± 1.39	4.50 ± 1.13	4.99	<0.001
DOP	3.97 ± 1.55	3.43 ± 1.56	4.51 ± 1.35	8.40	<0.001

DGBI: disorders of gut–brain interaction; HCs: healthy controls; ER: emotional reactivity; LST: low sensory threshold; EOE: ease of excitation; AES: esthetic sensitivity; PS: punishment sensitivity; DOP: depth of processing. Patients with DGBI exhibited significantly higher total scores on the Chinese Highly Sensitive Person Scale (HSPS-C) (*p* < 0.001) and consistently elevated scores across all SPS subdimensions than HCs (all *p* < 0.001).

### 3.7 Group comparison of HSP

To investigate the distributional heterogeneity of HSP within DGBI populations, this study adopted the normative threshold established by Lionetti et al., defining the HSP cutoff as the 30th percentile (HSPS-C score ≥4.67) within the total sample distribution. Intergroup HSP proportions were analyzed using 2 × 2 contingency tables, with chi-squared testing revealing significantly elevated HSP proportions in DGBI patients compared to HCs (χ^2^ = 38.45, *p* < 0.001; Table 9).

**Table 9:** Group comparison of HSP after matching.

	n-HSP (%)	HSP (%)	*χ^2^*	*p*
HC	210 (82.7)	44 (17.3)	38.45	<0.001
DGBI	146 (57.5)	108 (42.5)		

DGBI: disorders of gut–brain interaction, HCs: healthy controls, HSP: highly sensitive person, n-HSP: non-highly sensitive person. The χ^2^ analysis revealed a significantly higher prevalence of HSP in the DGBI group (42.5%) than in the HC group (17.3%) (χ^2^ = 38.45, *p* < 0.001).

### 3.8 Logistic regression analysis of risk factors for DGBI

#### 3.8.1 Univariate analysis

Using DGBI diagnosis as the dependent variable, binary logistic regression models were constructed with age, sex, dietary habits, BMI, alcohol consumption, and SPS subdimensions (ER, LST, EOE, AES, PS, and DOP) as independent variables.

A univariate analysis revealed that elevated scores in ER (OR = 1.629, 95%CI = 1.419–1.870), LST (OR = 1.553, 95%CI = 1.367–1.764), EOE (OR = 1.765, 95%CI = 1.532–2.034), AES (OR = 1.382, 95%CI = 1.213–1.574), PS (OR = 1.418, 95%CI = 1.229–1.637), DOP (OR = 1.644, 95%CI = 1.444–1.872), and alcohol consumption (OR = 4.885, 95%CI = 2.467–9.676) significantly increased DGBI risk (all *p* < 0.001). Conversely, a low-fat diet emerged as a protective factor against DGBI (*β* = −0.444, *p* = 0.013) (Table 10).

**Table 10:** Univariate logistic regression analysis of risk factors for DGBI after matching.

	*β*	OR (95%CI)	*p*
Age	0.011	1.011(0.985,1.038)	0.423
Sex	0.111	1.117(0.788,1.583)	0.534
ER	0.488	1.629(1.419,1.870)	<0.001
LST	0.440	1.553(1.367,1.764)	<0.001
EOE	0.568	1.765(1.532,2.034)	<0.001
AES	0.323	1.382(1.213,1.574)	<0.001
PS	0.349	1.418(1.229,1.637)	<0.001
DOP	0.497	1.644(1.444,1.872)	<0.001
Alcohol	1.586	4.885(2.467,9.676)	<0.001
Diet	−0.444	0.642(0.452,0.911)	0.013
BMI	0.073	1.076(1.005,1.152)	0.036

Univariate analysis revealed that elevated scores in ER, LST, EOE, AES, PS, DOP, and alcohol consumption significantly increased DGBI risk (all *p* < 0.001). Conversely, a low-fat diet emerged as a protective factor against DGBI (*β* = −0.444, *p* = 0.013).

#### 3.8.2 Multivariate model construction

Variables showing univariate significance were included in a multivariate logistic regression model. To address multicollinearity, variance inflation factors (*VIF*) were calculated, with a *VIF* >10 indicating severe collinearity (19). The analysis revealed acceptable collinearity across all predictors (*VIF* range: 1.016–2.953). The multivariate analysis incorporating all significant univariate predictors identified EOE (OR = 1.403, 95%CI = 1.110–1.773), DOP (OR = 1.315, 95%CI = 1.044–1.657), elevated BMI (OR = 1.110, 95%CI = 1.026–1.201), and alcohol consumption (OR = 5.258,95%CI = 2.480–11.150) as independent risk factors for DGBI (*p* < 0.05) (Table 11).

**Table 11:** Multivariate logistic regression analysis of risk factors for DGBI after matching.

	*β*	OR (95%CI)	*VIF*	*p*
ER	0.133	1.142 (0.898,1.452)	2.704	0.279
LST	0.003	1.003 (0.793,1.270)	2.953	0.978
EOE	0.339	1.403 (1.110,1.773)	2.485	0.005
AES	−0.056	0.946 (0.768,1.163)	2.032	0.596
DOP	0.274	1.315 (1.044,1.657)	2.841	0.020
PS	−0.045	0.956 (0.766,1.193)	1.938	0.690
Alcohol	1.660	5.258 (2.480,11.150)	1.030	<0.001
Diet	−0.351	0.704 (0.475,1.045)	1.016	0.082
BMI	0.104	1.110 (1.026,1.201)	1.046	0.010

OR: odds ratio; CI: confidence interval; DGBI: disorders of gut–brain interaction. Elevated scores in EOE and DOP, higher BMI, and alcohol consumption emerged as independent risk factors for DGBI. The multivariate analysis incorporating all significant univariate predictors identified EOE (OR = 1.403, 95%CI = 1.110–1.773), DOP (OR = 1.315, 95%CI = 1.044–1.657), elevated BMI (OR = 1.110, 95%CI = 1.026–1.201), and alcohol consumption (OR = 5.258, 95%CI = 2.480–11.150) as independent risk factors for DGBI (*p* < 0.05).

### 3.9 Predictive efficacy of SPS subdimensions for DGBI development

**Figure 5:**
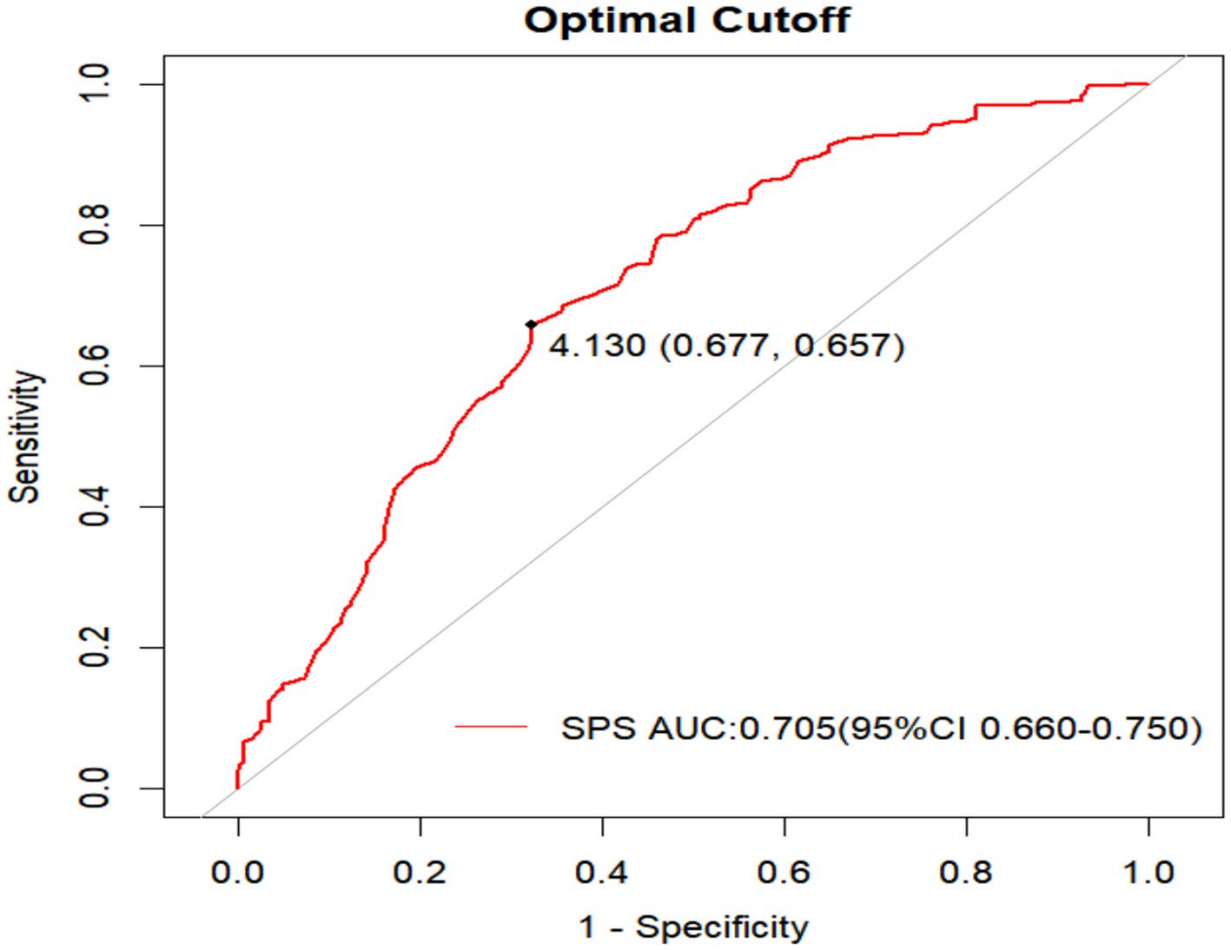
ROC curves of SPS in predicting DGBI.

A receiver operating characteristic (ROC) curve analysis was conducted to assess the discriminative efficacy of SPS for DGBI. The results showed moderate accuracy in identifying DGBI cases, with an area under the curve (AUC) of 0.705 (95% CI: 0.660–0.750). Using the Youden index method, the optimal cutoff value of 4.13 achieved balanced diagnostic efficacy, yielding a sensitivity of 65.7% and a specificity of 67.7% (Figure 5).

### 3.10 Comparison of SPS levels and HSP proportions between upper and lower gastrointestinal DGBI subgroups

To investigate whether DGBI patients with different symptom locations differ in their levels of Sensory Processing Sensitivity (SPS) and the distribution of Highly Sensitive Persons (HSP), this study categorized patients into two groups based on anatomical location: those with conditions above the ligament of Treitz were classified as the upper gastrointestinal DGBI group (U-DGBI, *n* = 123), while those with conditions below the ligament of Treitz were classified as the lower gastrointestinal DGBI group (L-DGBI, *n* = 131). Independent samples t-tests were used to compare differences between the U-DGBI and L-DGBI groups. The results indicated no statistically significant differences between upper and lower gastrointestinal DGBI patients in HSPS-C total scores (*t* = 0.41, *p* = 0.683), scores across subdimensions (*p* > 0.05), or the proportional distribution of HSP (*χ*² = 0.03, *p* = 0.859) (Tables 12, 13).

**Table 12:** Comparison of HSPS-C total and subscale scores between upper and lower gastrointestinal DGBI patients.

	All (*N* = 508)	U-DGBI (*N* = 123)	L-DGBI (*N* = 131)	*t*	*p*
HSPS-C	4.36 ± 1.06	4.33 ± 1.03	4.39 ± 1.10	0.41	0.683
ER	4.35 ± 1.29	4.36 ± 1.23	4.34 ± 1.37	0.16	0.875
LST	4.10 ± 1.41	4.10 ± 1.35	4.09 ± 1.49	0.05	0.964
EOE	4.08 ± 1.34	3.98 ± 1.30	4.20 ± 1.40	1.28	0.203
AES	4.77 ± 1.25	4.73 ± 1.26	4.80 ± 1.24	0.45	0.656
PS	4.50 ± 1.13	4.48 ± 1.13	4.52 ± 1.14	0.29	0.775
DOP	4.51 ± 1.35	4.46 ± 1.35	4.57 ± 1.35	0.67	0.506

**Table 13:** Comparison of HSP proportions between upper and lower gastrointestinal DGBI patients.

	n-HSP (%)	HSP (%)	*χ^2^*	*p*
U-DGBI	70 (56.9)	53 (43.1)	0.03	0.859
L-DGBI	76 (58.0)	55 (42.0)		

## 4 Discussion

This study is the first to use the Chinese version of the Highly Sensitive Person Scale (HSPS-C) in a population with disorders of gut–brain interaction (DGBI). By applying propensity score matching (PSM) to control for confounders such as age, sex, and education level, it systematically compared the levels of sensory processing sensitivity (SPS) and the distribution of highly sensitive persons (HSPs) between DGBI patients and healthy controls. Furthermore, the study extended its analysis to compare SPS levels and HSP proportions across different DGBI subtypes categorized by anatomical symptom localization. The discriminative efficacy of SPS for identifying DGBI was also evaluated.

The results of this study demonstrated that the HSPS-C has good psychometric properties in the investigated population, effectively capturing the multidimensional characteristics of sensory processing sensitivity (SPS). After PSM, patients with DGBI showed significantly higher total SPS scores and a markedly higher prevalence of highly sensitive persons (HSPs) than healthy controls. Among the SPS subdimensions, ease of excitation (EOE) and depth of processing (DOP) emerged as independent predictors of DGBI. Notably, no significant differences in SPS levels or HSP distribution were observed between upper gastrointestinal DGBI (U-DGBI) and lower gastrointestinal DGBI (L-DGBI) subtypes across different anatomical locations. Furthermore, overall SPS showed a moderate discriminative capacity for identifying DGBI. Collectively, these findings provide new empirical evidence for elucidating the psychophysiological mechanisms underlying DGBI and support the implementation of stratified clinical management strategies.

Propensity score matching serves as a critical methodology in observational study designs to control confounding bias. Its core principle involves constructing a composite score from multidimensional covariates to simulate the intergroup balance of randomized controlled trials, thereby enhancing the validity of causal inference (20). Previous studies have shown that women exhibit significantly higher SPS levels than men (21–23), with additional age-related variations in SPS profiles (24, 25). Furthermore, educational attainment may shape environmental adaptation strategies among highly sensitive individuals through disparities in cognitive resources and social support. These covariates were therefore incorporated into the propensity score matching framework to eliminate their confounding effects.

Psychosocial factors are intricately associated with DGBI patients’ illness awareness, symptom articulation difficulties, clinical severity, and outcomes (26). Research has indicated significant disparities in symptom duration, severity, and functional impairment among irritable bowel syndrome (IBS) patients with distinct personality profiles (27). Our findings of elevated SPS subdimension scores in DGBI patients align with Takahashi et al.’s (28) Japanese validation study of the HSPS, which reported significant correlations between gastrointestinal symptoms and LST (*r* = 0.24) and EOE (*r* = 0.29). The multivariate logistic regression analysis identified EOE (OR = 1.403, 95%CI = 1.110, 1.773), DOP (OR = 1.315, 95%CI = 1.044, 1.657), elevated BMI (OR = 1.110, 95%CI = 1.026, 1.201), and alcohol consumption (OR = 5.258, 95%CI = 2.480, 11.150) as independent DGBI risk factors. Each one-unit increase in average EOE and DOP scores was associated with increased odds of having DGBI by 40.3 and 31.5%, respectively. Neuroimaging evidence has revealed that HSPs exhibit heightened activation in the precuneus, prefrontal cortex, inferior frontal gyrus, and amygdala during stimulus exposure compared to non-HSP (29–31), suggesting a potential neurobiological link in which core SPS dimensions are associated with alterations in visceral sensitivity and gastrointestinal motility, potentially contributing to the observed susceptibility in DGBI (32).

Notably, our subgroup analysis further revealed no statistically significant differences in total SPS scores, scores across its subdimensions, or the distribution of HSP between patients with upper gastrointestinal DGBI (U-DGBI) and those with lower gastrointestinal DGBI (L-DGBI). This finding provides strong support for the hypothesis of a “shared psychophysiological origin” within the DGBI spectrum as a whole. SPS, as a stable trait reflecting the depth of processing and reactivity of the central nervous system to environmental stimuli, likely exerts its influence primarily on higher integrative centers of the brain–gut axis (such as the insula, anterior cingulate cortex, and prefrontal cortex) and associated autonomic regulatory pathways, rather than targeting a specific gastrointestinal segment. Therefore, regardless of whether symptoms are primarily localized to the upper or lower GI tract, the common underlying psychophysiological basis—namely, central sensory amplification and dysregulation—may be similar. This aligns perfectly with the core rationale of the Rome IV criteria, which redefines these disorders as “disorders of gut–brain interaction” rather than purely organic diseases (1). Furthermore, these results suggest that future research should move beyond traditional anatomical classifications and focus more on identifying endophenotypes based on underlying mechanisms (e.g., central sensitization, autonomic function, and specific psychological traits). Such an approach may be more instructive for developing transdiagnostic, targeted treatments, such as neuromodulation therapies or specific psychological interventions.

The multivariable logistic regression analysis identified elevated BMI (OR = 1.110) and alcohol consumption (OR = 5.258) as independent risk factors for DGBI. Pathophysiologically, obesity may contribute to DGBI, particularly functional dyspepsia, through mechanisms including leptin-mediated suppression of gastrointestinal motility (33, 34) and gut dysbiosis-associated visceral hypersensitivity (35, 36). Alcohol may disrupt gastrointestinal function bidirectionally by impairing the gastric mucosal barrier, altering acid secretion, and affecting motility (37). Notably, treating alcohol consumption as a binary variable rather than a dose–response variable might have inflated the estimated odds ratio. Although a low-fat diet appeared protective in the univariable analysis, its association lost significance after multivariable adjustment, suggesting potential confounding. The relationship thus requires clarification in studies with stricter confounder control. While prior studies linked smoking to specific conditions like functional abdominal pain (38) and IBS (39, 40), but not to functional constipation (41) or dyspepsia (42). We found no significant association between smoking and overall DGBI, which may be attributable to our inclusion of multiple DGBI subtypes potentially obscuring a subtype-specific effect within this heterogeneous patient population.

Receiver operating characteristic curve analysis evaluating the ability of SPS to distinguish DGBI cases from healthy controls revealed moderate discriminative efficacy, with an area under the curve (AUC) of 0.705 (95% CI: 0.660–0.750). At the optimal HSPS-C cutoff score of >4.13 (determined via Youden Index maximization), sensitivity and specificity reached 65.7 and 67.7%, respectively. This discriminative performance may be attributable to the multidimensional nature of SPS, where subdimensions such as ER and LST could collectively contribute to its association with DGBI via distinct neurobiological pathways. However, using SPS alone yielded non-negligible false-negative (34%) and false-positive (32%) rates, highlighting the need for integrated prediction models that combine SPS with biomarkers (e.g., serum 5-HT levels and gut microbiota diversity) and clinical indices (e.g., anxiety scores and stress exposure history) to enhance diagnostic precision.

Despite limited standalone discriminative power, SPS retains clinical value as a cost-effective, non-invasive screening tool for DGBI risk stratification. Potential applications include: Primary Prevention: Identifying high-SPS individuals for targeted lifestyle interventions (e.g., mindfulness-based stress reduction and environmental modulation). Personalized Treatment: Guiding therapeutic strategies based on SPS profiles in diagnosed patients. Psychosomatic Integration: Validating the biopsychosocial model by bridging psychological traits with gastrointestinal pathophysiology. Furthermore, the SPS-DGBI association provides empirical support for the psychosomatic medicine framework, advocating for clinicians to incorporate psychological trait assessments into routine practice rather than focusing solely on biological approaches to symptom management.

This study confirms a significant association between sensory processing sensitivity (SPS) and disorders of gut–brain interaction (DGBI), providing new evidence for the pathological link between SPS traits and somatic symptom disorders. However, the mechanisms underlying HSPs’ susceptibility to gastrointestinal diseases or symptoms remain incompletely understood. Potential explanations include: 1. Stress Sensitivity: Compared to the general population, HSPs are more prone to perceiving stress and being affected by negative environmental factors (43). Stress can alter visceral sensitivity and gut microbiota via the brain–gut axis, contributing to DGBI development (44, 45). 2. LST: HSPs exhibit heightened sensitivity to subtle stimuli (46), which may increase their awareness of minor abdominal discomfort or pain. Our findings, which identified LST as an independent risk factor for DGBI, support this hypothesis. 3. Genetic Links: Studies suggest that SPS is associated with polymorphisms in the serotonin transporter gene-linked polymorphic region (5-HTTLPR), with higher SPS levels associated with the short allele genotype (S/S) (47). The S/S genotype reduces serotonin transporter (SERT) expression, leading to decreased serotonin reuptake and elevated plasma serotonin levels (48, 49). Serotonin (5-HT), an indole derivative widely distributed in the central nervous and gastrointestinal systems, is primarily produced by enterochromaffin cells (ECs) in the gut. Approximately 95% of the body’s serotonin resides in the digestive tract, with 90% localized to ECs (50). Elevated serotonin levels may be involved in DGBI pathophysiology through alterations in visceral sensitivity, gastrointestinal motility, and intestinal permeability (51). These pathways likely interact within a “gene–brain–gut–microbiome” network. For instance, the S/S genotype may epigenetically enhance hypothalamic–pituitary–adrenal (HPA) axis reactivity, which in turn can synergistically modulate gut microbiota composition and serotonin metabolism, collectively contributing to DGBI pathogenesis.

Strengths of the study: 1. This is the first study to explore the association between SPS and DGBI and quantify the predictive value of SPS subdimensions. Our findings highlight that SPS is not only associated with mental health but also associated with physical health. 2. All DGBI patients were diagnosed after excluding organic pathologies via gastrointestinal endoscopy, rather than relying solely on ROME IV questionnaires. 3. This study implemented a standardized psychological assessment protocol to conduct rigorous screening of all participants. Through clinical interviews combined with multidimensional diagnostic tools, we systematically excluded individuals with psychiatric comorbidities. The screening process deliberately avoided sole reliance on scale-based instruments for evaluation and diagnosis. This methodological design not only aligns with the gold standard of clinical diagnosis but also effectively controls participants’ cognitive load, thereby enhancing the scientific validity and reliability of research outcomes. 4. PSM effectively balanced baseline characteristics between the groups, minimizing confounding bias and strengthening the validity.

Limitations of the study: 1. The cross-sectional design precludes the causal interpretation of SPS-DGBI relationships. Longitudinal cohort studies are needed to clarify temporal dynamics. 2. Potential selection bias occurred due to single-center patient enrollment. 3. Questionnaire-based data collection may be susceptible to recall bias or social desirability effects. 4. The diagnosis in this study was primarily based on the Rome IV clinical symptom criteria, with organic diseases excluded via endoscopy; however, comprehensive digestive physiological assessments were not routinely performed. For example, anorectal manometry was not conducted in constipated patients to objectively evaluate anal sphincter function and rectal sensory thresholds. Furthermore, the study did not use gastrointestinal symptom-specific psychometric instruments, such as the Esophageal Hypervigilance and Anxiety Scale or the Visceral Sensitivity Index, which could more precisely elucidate the potential links between sensory processing sensitivity and psychological distress associated with gastrointestinal symptoms. Therefore, future research integrating more complete physiological examinations and symptom-specific psychological assessments would enhance the reliability and clinical applicability of the conclusions. 5. DGBI encompass diverse subtypes (e.g., IBS and functional dyspepsia), and heterogeneity across subtypes may influence study findings. 6. The present study primarily included clinically common subtypes (functional dyspepsia and irritable bowel syndrome), while less common DGBI requiring specialized diagnostic tests, (e.g., certain functional esophageal disorders or defecatory disorders) were not sufficiently represented. Consequently, the generalizability may be somewhat limited. Future research could involve conducting multicenter, large-sample clinical studies that systematically enroll patients across all DGBI categories under the Rome IV classification to further validate the universality and specificity of SPS across all DGBI subtypes.

## 5 Conclusion

This study implemented propensity score matching (PSM) to control for confounding variables, conducting the first systematic investigation into the association between sensory processing sensitivity (SPS) and disorders of gut–brain interaction (DGBI). The research validated the clinical utility of the Highly Sensitive Person Scale-Chinese version (HSPS-C) in Chinese populations, quantified the discriminative capacity of SPS for DGBI identification, and highlighted the imperative to integrate SPS assessment into clinical frameworks. These findings provide novel perspectives for early DGBI screening and personalized interventions.

## Data Availability

The original contributions presented in the study are included in the article/supplementary material, further inquiries can be directed to the corresponding author/s.

## Glossary

DGBI: disorders of gut–brain interaction
FD: functional dyspepsia
IBS: irritable bowel syndrome
SPS: sensory processing sensitivity
HSP: highly sensitive persons
PSM: propensity score matching
ROC: receiver operating characteristic
ER: emotional reactivity
LST: low sensory threshold
EOE: ease of excitation
AES: esthetic sensitivity
PS: punishment sensitivity
DOP: depth of processing
HSPS-C: The Chinese Highly Sensitive Person Scale
HCs: healthy controls
CFA: confirmatory factor analysis
RMSEA: root mean square error of approximation
TLI: Tucker–Lewis index
NFI: normed fit index
CFI: comparative fit index
VIF: variance inflation factors
OR: odds ratio
CI: confidence interval
